# Genetic correlates of psychological responses to the COVID-19 crisis in young adult twins in Great Britain

**DOI:** 10.21203/rs.3.rs-31853/v1

**Published:** 2020-05-27

**Authors:** Kaili Rimfeld, Margherita Malancini, Andrea Allegrini, Amy E. Packer, Andrew McMillan, Rachel Ogden, Louise Webster, Nicholas G. Shakeshaft, Kerry L. Schofield, Jean-Baptiste Pingault, Argyris Stringaris, Sophie von Stumm, Robert Plomin

**Affiliations:** King’s College London, Social, Genetic and Developmental Psychiatry, Institute of Psychiatry, Psychology & Neuroscience; King’s College London, Social, Genetic and Developmental Psychiatry, Institute of Psychiatry, Psychology & Neuroscience; King’s College London, Social, Genetic and Developmental Psychiatry, Institute of Psychiatry, Psychology & Neuroscience; King’s College London, Social, Genetic and Developmental Psychiatry, Institute of Psychiatry, Psychology & Neuroscience; King’s College London, Social, Genetic and Developmental Psychiatry, Institute of Psychiatry, Psychology & Neuroscience; King’s College London, Social, Genetic and Developmental Psychiatry, Institute of Psychiatry, Psychology & Neuroscience; King’s College London, Social, Genetic and Developmental Psychiatry, Institute of Psychiatry, Psychology & Neuroscience; King’s College London, Social, Genetic and Developmental Psychiatry, Institute of Psychiatry, Psychology & Neuroscience; King’s College London, Social, Genetic and Developmental Psychiatry, Institute of Psychiatry, Psychology & Neuroscience; Clinical, Educational & Health Psychology, Division of Psychology & Language Sciences, Faculty of Brain Sciences, University College London; Mood, Brain & Development Unit, Emotion and Development Branch, National Institute of Mental Health; Psychology in Education Research Centre, Department of Education, University of York; King’s College London, Social, Genetic and Developmental Psychiatry, Institute of Psychiatry, Psychology & Neuroscience

**Keywords:** COVID-19, lockdown, psychological and behavioural traits, twins, young adults, England and Wales

## Abstract

We investigated how the COVID-19 crisis and the extraordinary experience of lockdown affected young adults in England and Wales psychologically. One month after lockdown commenced (T2), we assessed 30 psychological and behavioural traits in 4,000 twins in their mid-twenties and compared their responses to the same traits assessed in 2018 (T1). Mean changes from T1 toT2 were modest and inconsistent: just as many changes were in a positive as negative direction. Twin analyses revealed that genetics accounted for about half of the reliable variance at T1 and T2. Genetic factors correlated on average .86 between T1 and T2 and accounted for over half of the phenotypic stability. Systematic environmental influences had negligible impact on T1, T2 or T2 change. Rather than the crisis fundamentally changing people psychologically, our results suggest that genetic differences between individuals play a fundamental role in shaping psychological and behavioural responses to the COVID-19 crisis.

## Background

It is rare for such massive and abrupt social change to occur as the world has experienced with the COVID-19 pandemic and lockdown. COVID-19 disease can be a life or death issue for those infected with the virus, but the psychological responses of those infected and of the many more people in lockdown who had not contracted the disease are also of concern. For example, a recent review of 24 studies on the effects of quarantine concluded that ‘the psychological impact of quarantine is wide-ranging, substantial, and can be long lasting’ (Brooks et al., 2020). Low mood and irritability stood out with an incidence of 73% and 57%, respectively, but negative effects were also found for diverse measures including stress, anxiety and insomnia, with some indication of long-term effects such as post-traumatic stress and drug abuse. Also, many studies have found increased posttraumatic stress symptoms following natural disasters such as earthquakes and man-made disasters such as terrorism (Furr et al., 2010).

This and other research (Galea et al., 2020) suggests that the COVID-19 pandemic will worsen psychological health on average in a population. However, the crisis is likely to affect individuals differently, possibly even including some people whose psychological health is improved by the crisis. An important issue is that the causes of mean differences can be unrelated to the causes of individual differences. For example, the cause of mean changes before and after the COVID-19 crisis can safely be attributed to the environmental effects of the pandemic and lockdown. However, this does not imply that differences in pandemic experiences are the sole source of individual differences in response to the crisis. Importantly, the way in which individuals react to the same event can depend on their genetics.

Here we investigated genetic as well as environmental influences on individual differences in psychological and behavioural traits before the COVID-19 crisis and lockdown (T1) and one month after lockdown had commenced in the UK (T2). In addition to asking participants how they think the crisis affected them, we compared the same psychological and behavioural traits obtained at T1 and T2 on the same individuals with data at both T1 and T2. To assess the aetiology of individual differences, we used the classical twin design based on the resemblance of identical and non-identical twins. From 17 April to 4 May 2020, we collected online data from 4000 twins in our Twins Early Development Study (TEDS; Rimfeld et a1., 2019) from whom we already had data at T1. We included 30 diverse psychological constructs, such as anxiety, depression, well-being, alcohol use, relationships, achievement motivation, purpose in life, life goals, physical activity, online behaviour, volunteering, and community satisfaction. These same measures had been included in a 2018 wave of assessment in TEDS (T1).

The twins were born between 1994 and 1996. They were thus in their early twenties during T1 and T2. Few twin studies have focused on this age when the twins are completing their studies and beginning their adult life, entering the workforce, and forming long-term relationships. At this tipping point in their lives, it could be argued, have the most to lose from the crisis personally, socially and economically.

We describe mean changes from T1 to T2, hypothesizing that changes will be modest and inconsistent, with some positive as well as negative changes (Hypothesis 1). However, our focus is on individual differences and their genetic and environmental origins at T1 and T2 and in changes from T1 to T2. On the assumption that the COVID-19 crisis affected people differently, we hypothesised that variance will be greater at T2 than T1 (Hypothesis 2). We predicted that phenotypic correlations will be substantial between T1 and T2 (Hypothesis 3).

Our overall hypothesis is that genetics, by which we mean inherited DNA differences, is the major systematic force governing how people respond psychologically to the COVID-19. Specifically, we expected that all traits will show substantial genetic influence at T1, as indicated by a large body of genetic research on psychological traits (Knopik et al., 2017; Polderman et al., 2015). We also hypothesised that, despite the crisis, genetics will be similarly influential at T2 (Hypothesis 4). We also predicted that heritability will be

Genetic correlates of psychological responses to COVID-19 lower for T1 to T2 change scores because they only capture genetic effects at T2 that are independent of genetic effects at T1. We operationalised change by regressing T1 scores from T2 scores so that T2 scores are independent of scores at T1, which we refer to as ‘change scores’. Crucially, we predicted that genetic correlations between T1 and T2 will be substantial (Hypothesis 5), indicating that, despite the COVID-19 crisis, individual differences at T2 are largely governed by the same genetic factors that affect T1.

Environmental factors are important too, but we predicted that their effects on individuals are not the systematic effects of family environment. The twin design can be used to distinguish systematic environmental influences attributable to growing up in the same family, called ‘shared’ environmental influences, from other environmental influences (Plomin & Daniels, 1987). Despite a century of the ‘nurture assumption’ in which the family was assumed to be the major systematic source of environmental influence (Harris, 1998), shared environmental influences are generally negligible, and especially as young adults leave their family and make their own way in the world (Plomin, 2018). This is the rationale for our hypothesis the such shared environmental influences will have negligible impact at both T1 and T2 as well as for change from T1 to T2 (Hypothesis 6). Although environmental effects are substantial, our hypothesis is that the environmental effects that make a difference are largely ‘non-shared’, idiosyncratic and unsystematic (Plomin, 2018).

We predicted that similar results will be obtained from bivariate model-fitting analysis (Hypothesis 7). That is, most of the genetic effects on T2 scores will be accounted for by genetic effects in common with T1, although there will be some novel genetic effects at T2 independent of T1. Environmental effects due to shared rearing or living circumstances during lockdown will be negligible.

Finally, we predicted that these results for T2 change will not interact significantly with potential moderators (Hypothesis 8). Lockdown presents a quasi-experimental test of contemporary shared environments by comparing results for twins living together during lockdown and those living apart. If such shared experiences were important, twins locked down together should be more similar than twins living apart during lockdown. On the basis of the generally weak effects of shared rearing environment, we predicted that environmental effects due to living together during lockdown will be negligible. We also investigated other possible moderators of genetic and environmental influences on individual differences in psychological traits before and during the COVID-19 crisis, such as conditions of lockdown, having COVID-19 symptoms, socioeconomic status and gender, although power to detect such interactions is limited.

All of our hypotheses were preregistered prior to analysis with Open Science Framework: https://osf.io/r58be/.) In summary, they were:

1. Mean changes from T1 to T2 will be modest and
2. Variance will be greater at T2 than
3. Phenotypic correlations will be substantial between T1 and
4. Heritability of individual differences will be substantial for all traits at T1 and T2. Heritability will be lower for T1 to T2 change
5. For all traits, genetic correlations between T1 and T2 will be
6. Environmental influences due to shared rearing or current living circumstances will be negligible for all traits at T1 and T2 as well as for T2 change
7. Similar results will be obtained from a bivariate model-fitting analysis across T1 and
8. These results for T2 change will not interact significantly with potential moderators.

## Results

### Means

**Figure 1.**
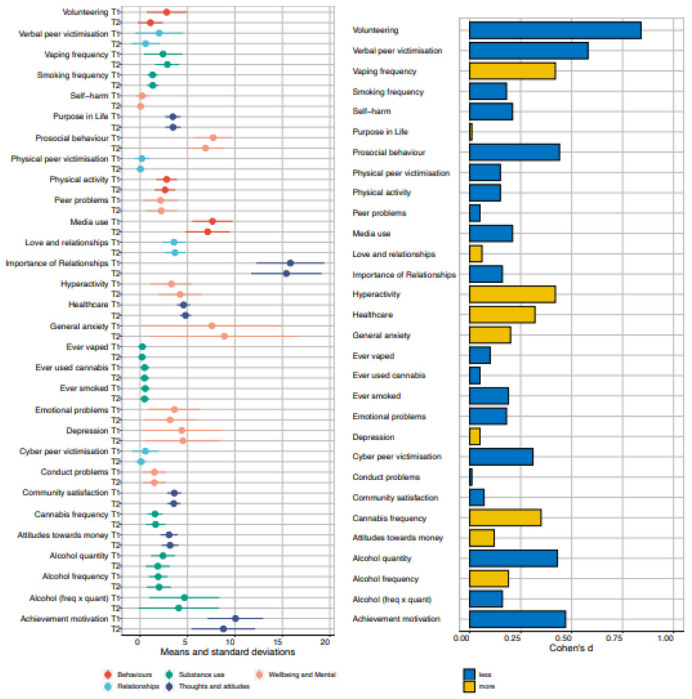
Descriptive statistics for all measures at T1 and T2. Means and standard deviations for all the measures are presented in the panel on the left. On the right are effect sizes (Cohen’s d) for the differences between phenotypes at T1 and T2.

Figure 1 illustrates means and standard deviations for the 30 measures at T1, T2 and for T2 change. The details of the descriptive statistics, along with descriptive statistics further broken down by gender and zygosity, are included in Supplementary Tables 1–6. These results are based on one twin randomly selected from each pair so that the data points are independent. Results for the other twin are virtually identical, as shown in Supplementary Tables 7–9.

Almost as many changes were in a positive direction as in a negative direction. However, the effect sizes are modest as indicated by Cohen’s *d* statistic, which is the ratio of the mean difference to the standard deviation (Cohen, 1988; Figure 1). The average *d* across the 30 measures was 0.24, which accounts for less than two percent of the variance and includes as many positive as negative changes.

Cohen (1988) proposed, as convention, that a large effect size is a *d* of 0.8, accounting for about 25% of the variance. Only one large negative effect emerged, decreased Volunteering (0.84), which is almost certainly due to less opportunity for volunteering during lockdown.

A *d* of 0.5, considered a medium effect size, accounts for about 9% of the variance. Medium-sized mean differences in the negative direction emerged for three variables. Prosocial Behaviour declined (0.44), which, like Volunteering, might be due in part to reduced opportunity. Achievement Motivation decreased (0.47), which is worrying because emerging adults are our next generation of workers. Hyperactivity-Inattention increased (0.42), which seems to fit with reports in the media that people feel less able to concentrate. Other effect sizes were modest (*d* = 0.20).

### Variances

These mean differences mask a wide range of individual differences. If the COVID-19 crisis affected people in more extreme ways, we would expect to see increased variance at T2. The standard deviations (Supplementary Tables 1 and 2) do not support this hypothesis. The average standard deviation at T2 (1.71) was slightly *lower* than at T1 (1.79).

For these analyses and the following analyses of individual differences, we focused on variables that showed sufficient variability and approached normal distributions, including Achievement motivation, Alcohol, Community satisfaction, Conduct problems, Depression, Emotional problems, General anxiety, Healthcare, Hyperactivity/inattention, Importance of relationships, Love and relationships, Media use, Money attitudes, Peer problems, Physical activity, Prosocial behaviour, Purpose in life and Volunteering

### Covariances

**Figure 2.**
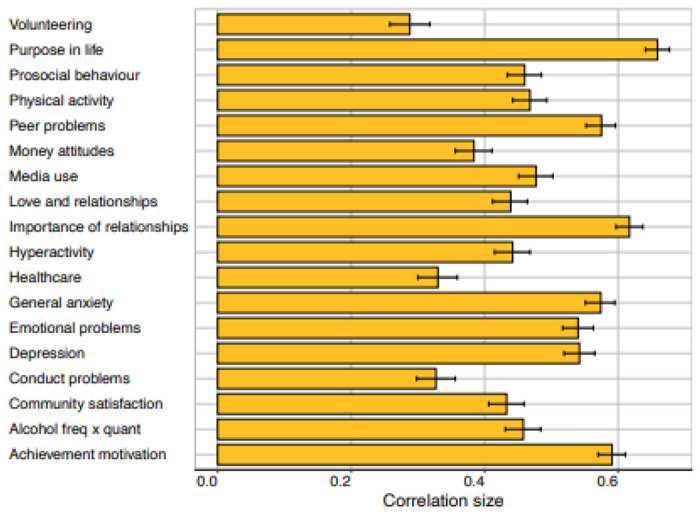
Phenotypic correlations (and 95% confidence intervals) between measures at T1 and T2.

If the COVID-crisis re-shuffled the rank order of individual differences, we would expect to see little stability from T1 to T2. Pearson correlations from T1 to T2 are shown in Figure 2 and listed in Supplementary Table 10, separately for males and females. The average correlation is 0.48 across the two-year gap. The most stable measures include Purpose in Life (0.68), Emotional Problems (0.56), Peer Problems (0.58), General Anxiety (0.57), and Depression (0.56). Stability correlations were generally similar for males and females, with average stability correlations of 0.50 and 0.47, respectively.

Reliability of the measures represents a ceiling for stability. In TEDS, we obtained two-week test-retest reliability from TEDS twins on most measures as part of our preparatory work for the 2018 (T1) assessment (Supplementary Table 11). The average test-retest reliability was 0.71, ranging from 0.47 for Importance of Healthcare to 0.84 for Volunteering. The average stability correlation of 0.48 implies that 48% of the *total* variance of the measures was stable from T1 to T2. Taking test-retest reliability into account (through dividing the correlation estimate by the test-retest coefficient) suggests that 68% of the *reliable* variance of the measures was stable from T1 to T2.

Despite the substantial stability from T1 to T2, T2 change scores revealed some individuals who changed dramatically in positive as well as negative directions, as illustrated in Supplementary Figure 1. We will assess the twins on the same measures on three more occasions during 2020, which will enable analyses of the perseverance and prediction of these extremes.

### Genetic and environmental aetiologies of variances and covariances

#### Twin correlations.

**Figure 3.**
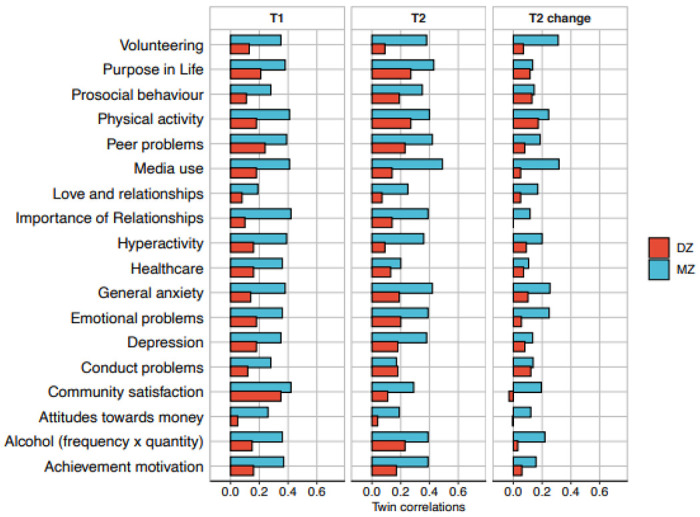
Correlations between MZ and DZ twin pairs for all measures at T1, T2 and T2 change.

Figure 3 depicts intraclass correlations for identical and non-identical twins at T1 and T2 and for T2 change scores. (See Supplementary Tables 12–14 for the correlation coefficients). We will describe the main results of the twin analysis using these twin correlations, although later we show that these results are confirmed by structural equation modelling, which also provides 95% confidence intervals for the genetic and environmental estimates.

At T1, the average twin correlations for identical and non-identical twins were 0.35 and 0.16, respectively. Because identical twins are identically genetically whereas non-identical twins are only 50% similar genetically, the difference in their correlations indexes genetic influence on individual differences, called heritability. Doubling the difference between these correlations suggests a rough estimate of heritability of 35% at T1 because heritability cannot exceed the identical twin correlation. At T2, the average twin correlations for identical and non-identical twins were similar, 0.31 and 0.16, as was the average heritability of 30%, despite the COVID-19 crisis and lockdown.

Twin resemblance not explained by zygosity can be attributed to shared environment (C). In other words, the extent to which heritability does not account for the identical twin correlation is a rough index of C. On average, C was negligible at T1 (2%) and T2 (4%).

The rest of the variance is attributed to a residual component of variance (E) that includes non-shared environment plus unreliability of measurement. The average E was 63% at T1 and 66% at T2. Test-retest reliabilities suggest that non-shared environment accounted for about half of E at T1 and T2.

Deducting the component of variance due to unreliability indicates that about half of the *reliable* variance at T1 and T2 can be attributed to inherited DNA differences. In other words, of the *total* variance at T1 and T2, about 40% can, on average across the measures, be attributed to genetic factors, about 30% to non-shared environmental factors, and about 30% to unreliability of measurement. Shared environmental influence has negligible impact.

T2 change scores show lower heritabilities, 16% on average. Because T2 change is a residualised score independent of scores at T1, stable genetic influence from T1 to T2 is removed from T2 change scores.

Thus, heritability of T2 change scores represents novel genetic influence at T2 that does not affect T1. Shared environment, which includes not only shared rearing environment (the twin pairs grew up together in the same family) but also shared experiences during the COVID-19 crisis, has negligible effects on T2 change, 3% on average. Most of the variance of T2 change scores is due to the E component of variance, 81% on average. We cannot separate E of T2 change scores into non-shared environment and unreliability of measurement because test-retest reliability at T1 cannot be assumed to represent the reliability of T2 change scores.

#### Univariate model-fitting results.

**Figure 4.**
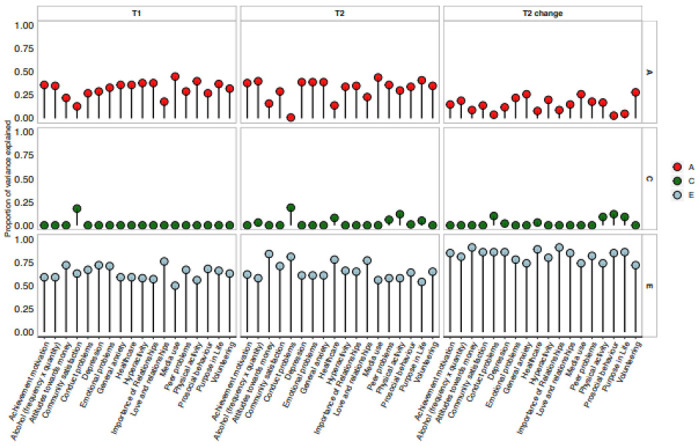
Univariate model-fitting estimates.

These results about variance and covariance gleaned from the twin correlations are highly similar to the results of univariate model-fitting analyses of variance for T1, T2 and T2 change measures, as shown in Figure 4. (See Supplementary Table 15 for model-fit statistics, precise ACE estimates and confidence intervals.) The average model-fitting heritability estimates were 32% for T1, 32% for T2 and 15% for T2 change. Model-fitting estimates of shared environment were 3% for T1 measures, 3% for T2 measures and 2% for T2 change measures. Average model-fitting estimates of E were 66%, 65% and 82%, respectively.

#### Bivariate model-fitting results.

**Figure 5.**
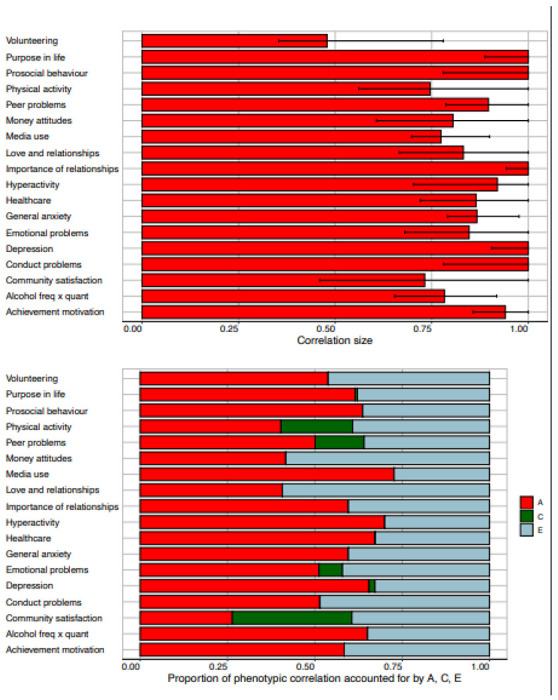
Bivariate model-fitting estimates. Genetic correlations are presented in the top panel. The bottom panel shows the proportion of the phenotypic correlation that is explained by A, C and E.

The Cholesky Decomposition bivariate model-fitting model separates A, C and E components of variance at T2 into variance in common with variance at T1 and variance at T2 independent of variance at T1. As explained in Methods, the model yields estimates of the extent to which the phenotypic correlation between T1 and T2 is accounted for by A, C and E. The genetic correlations are shown in the top panel of Figure 5 (See Supplementary Figure 2 for shared environmental and non-shared environmental correlations). The results of the Cholesky bivariate analysis are illustrated in the bottom panel of Figure 5, with details in Supplementary Tables 16–21. Genetics accounts for 55% of the T1-T2 phenotypic correlations on average. Shared environment accounts for 4% of the phenotypic correlations on average. E influences shared at T1 and T2 are responsible for the rest of the phenotypic correlations (40%), which could be stable non-shared environmental influences or correlated error.

The Cholesky model also estimates A, C and E components of variance at T2 independent of their respective A, C and E components of variance at T1. These A, C and E estimates of T2 change (Supplementary Tables 16–21) are, as expected, similar to the A, C and E estimates for T2 change shown in Figure 4.

Figure 5 also shows the genetic correlations between T1 and T2 and compares them to the phenotypic correlations described earlier. As explained in Analyses, the Cholesky model estimates the genetic contribution to phenotypic stability from T1 to T2, which includes the genetic correlation. The genetic correlation is the correlation between genetic effects at T1 and T2 independent of the T1 and T2 heritabilities. The genetic correlations averaged 0.91, and most of their 95% confidence intervals included 1.0, indicating that genetic effects at T2 were substantially correlated with genetic effects at T1, despite the COVID-19 crisis and lockdown.

#### Twins locked down together vs apart.

Finally, we investigated possible moderators of the univariate results. The most novel moderator is whether the twins were locked down together or living apart during lockdown. Lockdown presents a quasi-experimental test of contemporary shared environments by comparing results for the 28% of twins living together during lockdown and those living apart. If shared lockdown experiences were important, twins locked down together should be more similar than twins living apart during lockdown. On the basis of the generally weak effects of shared environment, we predicted that environmental effects due to living together during lockdown are negligible.

**Figure 6.**
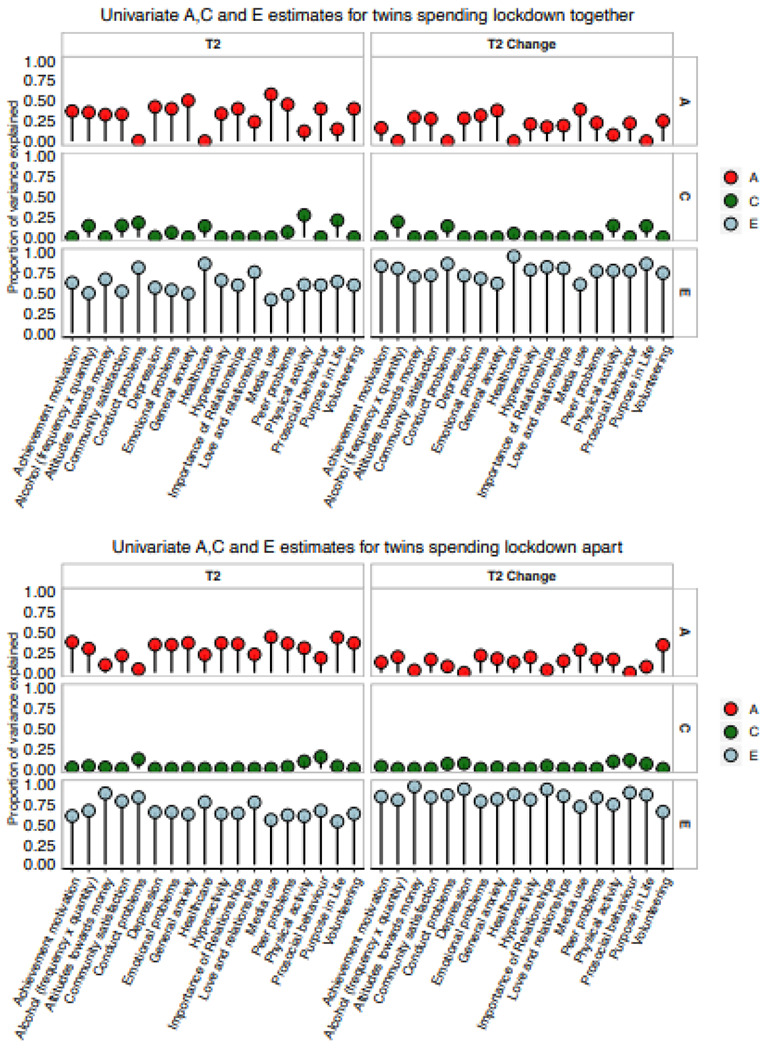
Univariate model-fitting estimates for twins in lockdown apart (top panel) vs. twins in lockdown together (bottom panel).

At first this prediction seemed wrong because the average twin correlation for twin pairs locked down together (.30) was higher than the correlation for twin pairs living apart during lockdown (.23), although this difference was not significant (p = .051). However, this possible effect of shared environments might be a genetic effect in disguise because identical twins locked down together more often than non-identical twins (32% vs 25%). Results of univariate model-fitting separately for twins locked down together vs apart (Figure 6) are consistent with the notion that the apparent effect of shared environments might be mediated in part genetically (Supplementary Table 26–27 for model-fitting results including the 95% confidence intervals). For T2 scores, twins together yielded a slightly higher average estimate of shared environmental influence compared to twins apart (.07 vs .03), suggesting some very slight increase in true shared environmental influence. However, twins together also yielded a slightly higher average estimate of genetic influence compared to twins apart (.33 vs .30), which could be the result of genetically influenced selection for being locked down together, which would be an example of gene-environment correlation. However, a great deal of caution is warranted in these interpretations because the difference in phenotypic correlations for twins locked down together vs apart is not significant and our design has negligible power to detect significant differences of this magnitude for A and C.

Power to detect significant differences for such small effects is negligible. Nonetheless, further support for the hypothesis that the apparent C effect of being locked down together is not really C comes from finding nearly identical A and C estimates pre-existing at T1: A and C are .33 and .06 for twins together and .30 and .03 for twins apart. Results of T2 change scores provides additional confirmation in that a similar pattern emerged: A and C are .19 and .04, respectively, for twins together and .14 and .02 for twins apart.

#### Other moderators.

We also considered other potential moderators. For example, similar to being locked down together or apart, gender is a dichotomous variable that is the same for both members of a twin pair (when opposite-sex non-identical twins are excluded). Separate univariate analyses for male and female twins yielded similar results. These model-fitting results are presented in Supplementary Tables 28 and 29.

For the continuous moderator of family SES and for moderators that can be discordant for members of a twin pair (living conditions during lockdown, COVID-19 symptoms, losing a job/financial difficulties), we corrected T2 and T2 change scores for these moderators and repeated the analyses. ACE estimates were similar when we compared estimates before and after correction for these moderators. These model-fitting results are included in Supplementary Table 30–36.

## Discussion

How much has the COVID-19 crisis changed young adults psychologically following the unprecedented social experiment of one month of lockdown? As expected, the 30 measures in our study yielded many statistically significant changes in means. The largest changes in the negative direction were reduced volunteering and achievement motivation and increased hyperactivity-inattention. However, there were as many changes in the positive direction, most notably, reduced verbal peer victimisation. Changes were similar in direction and magnitude for males and females, with the single exception of general anxiety, which increased more for females than males. However, most of these mean changes have modest effect sizes, with an average *d* of 0.24. Although we expected that the crisis would affect some individuals more than others, we found no increase in variance at T2. It is possible that the effects of the crisis will hit harder later or that longer lockdown or the economic aftermath of the crisis will have a greater effect. We will investigate these possibilities with three follow-up surveys during 2020.

Why do these young adults in Great Britain show modest negative effect on average after being in lockdown for one month when it is generally assumed that the psychological effects will be substantial? Part of the answer is that research often focuses on statistical significance and mean differences rather than considering effect size and individual differences. With our large sample size, nearly all variables show significant mean differences, but they don’t make much of a difference, accounting for less than two percent of the variance on average. Another reason might be methodological. In the present study we did not focus on participants’ subjective reports of how the COVID-19 crisis changed them. Instead, at T2, we asked participants to report, for example, how depressed they felt during the month following lockdown, which we compared to their reports of depression on the same measures in 2018. We found no difference in depression on average.

Other reasons why we found few negative effects of the COVID-19 crisis could be that the lockdown was so widespread (we’re all in it together spirit?) or that our participants are British (stiff upper lip?) or that they are young adults (resilience? insouciance?). Concerning the insouciance hypothesis, we asked participants at T2 how much they were worried about their physical health and mental health during the month since lockdown. The frequency of those reporting that they were moderately, very, or extremely worried was 38% for physical health and 57% for mental health. In other words, they were, quite reasonably, worried, although on average they did not change psychologically, including their symptoms of general anxiety. This can be viewed as a hopeful message that young people on average, are resilient psychologically to an experience as seismic as COVID-19 and lockdown, although these mean differences mask individual differences to the COVID-19 and lockdown. It remains to be seen if similar results emerge in other countries, at other ages and after longer exposure to the crisis and its aftermath.

The focus of our study was on individual differences rather than mean differences. How much has COVID-19 shuffled the deck of individual differences? The rank order of individual differences was largely stable from T1 to T2, with stability accounting for about 70% of the reliable variance at T1 and T2 on average across the measures. From a genetic perspective, the most interesting finding was that the average genetic correlation was 0.86, indicating that genetic effects at T1 were highly correlated with genetic effects at T2, despite the intervening COVID-19 crisis and lockdown. It is also interesting that T2 changes, which are independent of T1, show genetic influence.

We conclude that inherited DNA differences are the major systematic force shaping individual differences in psychological traits at T2 as well as at T1. Genetic effects account for about half of the reliable psychological differences between people at T1 and T2. The environment accounts for the rest of the variance, but it is not the systematic effect of environmental factors often assumed to be important, such as shared family environment. Environmental factors of this systematic sort had negligible effects on variance at T1 and T2 and for T2 change. The environmental effects that make a difference are those that are not shared by twin siblings growing up in the same family or, in our study, by twins locked down together. These idiosyncratic ‘non-shared’ environmental factors are likely to be unsystematic, chance experiences (Plomin, 2018).

Our results confirmed seven of our eight pre-registered (https://osf.io/r58be/) hypotheses. This speaks to the replicability of findings from behavioural genetic research on which these hypotheses were based, which is noteworthy given the replication crisis in science in general and in psychology in particular (Plomin et al., 2016). The exception was the hypothesis that variance at T2 would be greater than at T1, which was a prediction not based on behavioural genetic research. The consistency of results from T1 to T2 also attests to the replicability of research in behavioural genetics.

Concluding that inherited DNA differences are the major systematic force shaping who we are psychologically does not imply that novel environmental interventions, including therapeutic interventions, cannot make a difference. It should be emphasised that heritability does not imply immutability.

Heritability is a descriptive statistic limited to a particular population at a particular time with a particular mix of genetic and environmental influences. Our study can be seen as an attempt to assess whether heritability changed as a function of a tectonic shift in environment, the COVID-19 crisis.

Concluding that the COVID-19 crisis has not fundamentally changed these young people psychologically is not to dismiss the pain some of them felt before or during the crisis, or will continue to feel after the crisis ends. Even though the crisis had little effect on means and even less effect on variances and covariances, genetically driven psychological vulnerabilities are especially important targets for preventive interventions in young adults because the twenties is a pluripotent tipping point for life-long psychological problems (Arnett, 2014; Smith et al., 2011).

## Methods

### Sample

Our sample included young adult twins born in England and Wales between 1994 and 1996 enrolled in the Twins Early Development Study (TEDS; Rimfeld et al., 2019).TEDS recruited over 16,000 twin pairs at birth; more than 8,000 twin were invited to participate in TEDS’ 2018 assessment. Rich behavioural data have been collected from the twins developmentally over 14 waves of assessment in 20 years of data collection. Importantly, TEDS was a representative sample of the population in England and Wales at first contact, and remains reasonably representative in terms of family socioeconomic status and ethnicity (Rimfeld et al., 2019).

We used data collected when the twins were 21 to 24 years old (completed in 2018; T1) and data collected 17 April – 4 May 2020, approximately one month after the lockdown in response to the COVID-19 pandemic had started (T2). For the COVID-19 assessment, we only invited the subsample of twins for whom we had email addresses, which included many unpaired twins as well as pairs. The twins were invited by email and given a link and code to use to log on to the survey, a platform created and supported by Quodit Ltd. The survey began with an information sheet and consent mechanism. Incentives included a prize draw for iPads and shopping vouchers. Ethical approval was received from King’s College London Research Ethics Committee (Reference number PNM/09/10-104). Although 4 May was the cut-off used for the present analyses, we continued to collect data, which will be used in future papers.

For our analyses for this paper, we selected twins with data both at T1 and T2. Sample sizes for each measure at T1 and T2 is reported in Supplementary Tables 1 and 2. All available data were used in the analyses. The total sample size of individuals was 4000, which includes ‘unpaired’ twins in which data from only one member of a twin pair was available. The total number of twin pairs in which both members of a twin pair responded at both T1 and T2 was 1143. Of these twin pairs, 537 were pairs of identical twins, 365 were pairs of same-sex non-identical twins and 231 were pairs of opposite-sex non-identical twins. In order to increase the power of our twin analyses, we combined the two groups of non-identical twins using sex-corrected data, as described later.

The sample for the current data collection at T2 for whom we also have data at T1 remains reasonably representative of the population in England and Wales for some key demographic characteristics. For example, our sample was similar to UK equivalents (Rimfeld et al., 2019) for ethnicity (94% white vs 93%), father employed (94% vs 91%), and mother employed (47% vs 50%). However, the twins’ parents were somewhat more educated: father with A-levels or higher (54% vs 47%) and mother with A-levels or higher (46% vs 35%). The twins themselves were more likely to attend university (58% vs 42%), and they were also more likely to have completed three full courses of A-levels (58% vs 42%). Also, more females participated (63% vs 51%).

## Measures

The T1 assessment in TEDS, which surveyed twins when they were 21-24 years old, was completed in 2018. T1 data collection included a broad range of psychological measures such as wellbeing, thoughts and attitudes, relationships and behaviours of young adults, as well as measures of physical health (Figure 1a). These existing data provided us with a unique opportunity to examine how the COVID-19 crisis has changed the lives of young adults. The T2 data collection included the same measures that were collected at T1, as well as the **C**o**R**onav**I**ru**S** Health **I**mpact **S**urvey (CRISIS; Figure 1b), developed for the purpose of assessing the physical and psychological impact of COVID-19 (Marikangas & Stringaris, 2020). Data were collected using online questionnaires. The measures were administered online in an easy-to-use format created by Quodit and took 15 minutes on averaged to complete. Participants completed the study in web browsers, on their own computers or mobile devices. The measures included quality control items and other controls for inappropriate responses such as responding too quickly. Details about the measures and their references are included in Supplementary Table 11.

## Statistical analyses

Our statistical analysis plan was registered in the Open Science Framework, prior to creation of the dataset and prior to analysis (https://osf.io/r58be/).

### Phenotypic analyses

Means and standard deviations were calculated for all measures at T1, T2 and for the change between T1 and T2 (T2 change). Change scores were calculated by correcting T2 scores for T1 scores using the regression method (by rescoring the variable as a standardised residual correcting for T1). Descriptive statistics were calculated for the entire sample, and separately for males and females. Cohen’s d was used to obtain an estimate of the effect sizes of the mean differences (Cohen, 1988). Univariate analysis of variance (ANOVA) was used to investigate mean differences for males and females for T1, T2 and T2 change (Supplementary Tables 1–3). Because significant, though small, sex differences emerged, explaining 0-8 % of the variance in outcome measures, we corrected all scores for the mean sex differences using the regression method. Correcting for sex is important in the analysis of twin data members of a twin pair are identical in age and identical twins are identical for sex, and this would otherwise inflate twin estimates of shared environment (McGue & Bouchard, 1984). We also corrected the measures for variation in age.

Phenotypic correlations were calculated between T1 and T2 scores for the whole sample and for males and females separately as an index of stability. We then compared the stability to test-retest reliability that was obtained in 2018 prior to T1 data collection (Supplementary Table 11). In all phenotypic analyses, we included one, randomly selected, twin from each pair to account for the non-independence of observations in the sample (i.e. twin pairs). The results remained consistent when we examined the other randomly selected half of the sample (Supplementary Tables 7–9).

### Genetic analyses

#### Univariate twin analyses.

The twin design was used for univariate and bivariate genetic analyses. The twin method offers a natural experiment capitalising on the known genetic relatedness of identical (monozygotic, MZ) and non-identical (dizygotic, DZ) twin pairs. MZ twins are genetically identical and share 100% of their genes, while DZ twins share on average 50% of their segregating genes. Both MZ and DZ twins are assumed to share 100% of their shared environmental influences growing up in the same family. Non-shared environmental influences are unique to individuals, not contributing to similarity between twins. Using these known family relatedness coefficients, it is possible to estimate the relative contribution of additive genetic (A), shared environmental (C), and non-shared environmental (E) effects on the variance and covariance of the phenotypes, by comparing MZ correlations to DZ correlations. Heritability can be roughly calculated by doubling the difference between MZ and DZ correlations, C can be calculated by deducting heritability from MZ correlation and E can be estimated by deducting MZ correlation from unity (following Falconer’s formula) (Rijsdijk & Sham, 2002).

These parameters can be estimated more accurately using structural equation modelling, which also provides 95% confidence intervals and estimates of model fit. The structural equation modelling program OpenMx was used for all model-fitting analyses (Boker et al., 2011).

Here we present twin correlations and ACE estimates for T1, T2 and change scores for all variables. The difference in the intraclass correlations between MZ and DZ twin pairs can guide the decision of conducting an alternative to the ACE model, the ADE model. The ADE model partitions the variance into additive genetic (A), non-additive (or dominant) genetic (D) and non-shared environmental (E) effects. This model is fitted in cases when intraclass correlations for DZ twins are below 50% of the MZ intraclass correlation – indicating non-additive genetic influences. Although for a few traits the DZ correlation suggested the possibility of non-additive genetic effects, we opted for running ACE models across all variables for three key reasons: first, despite our large sample size, we lacked power to detect non-additive variance reliably; second, conducting the same models across all traits allowed us to meaningfully compare the results across all measures; third, even in studies equipped with the necessary power to detect non-additive genetic effects, it is rare to find a significant contribution of D (and C) for self-reported psychological traits measured in adulthood (Knopik, Neiderhiser, DeFries, & Plomin, 2017; Rijsdijk & Sham, 2002).

#### Bivariate twin analyses.

These univariate analyses can be extended to bivariate analyses to investigate the aetiology of covariance between two traits. Bivariate genetic method decomposes the covariance between traits into additive genetic (A), shared environmental (C), and non-shared environmental (E) components by comparing the cross-trait cross-twin correlations between MZ and DZ twin pairs. This method also enables estimation of the genetic correlation (*r*G), which is an index of pleiotropy, indicating the extent to which the same genetic variants influence two traits or measures of the same trait at two times. The shared environmental correlation (*r*C) and non-shared environmental correlation (*r*E) are estimated in a similar manner (Knopik, Neiderhiser, DeFries, & Plomin, 2017; Rijsdijk & Sham, 2002). We used bivariate genetic modelling to calculate rG, rC and rE between T1 and T2 measures.

In addition, we investigated possible moderation for the aetiology of individual differences in T2 and change scores following the COVID-19 lockdown. For dichotomous moderators that are the same for both members of twin pairs (i.e., twins locked down together versus apart and gender), we calculated ACE estimates separately for each group and compared the univariate ACE estimates between groups. For continuous moderators (SES) and for moderators that were mostly discordant for members of twin pairs (e.g. family socioeconomic factors, COVID-19 symptoms, losing a job/financial difficulties, living conditions during lockdown), we corrected the trait scores for the moderator using the regression method and repeated the analyses. We then compared the univariate ACE estimates before and after the correction.

#### Bivariate Cholesky decomposition.

The Cholesky decomposition (see Supplementary Figure 3) allows for examination of common and independent genetic (A), shared environmental (C) and non-shared environmental (E) effects on the covariance of two or more traits (Neale, Boker, Bergeman, & Maes, 2005). The model assesses the independent contribution of a predictor variable to the variance in the outcome variable, after accounting for the variance accounted for by other predictors. As illustrated by the path diagram in Supplementary Figure 3, the genetic and environmental variance in the outcome (y) is calculated after accounting for the variance that is explained by the predictor previously entered in the model (x). As for hierarchical regression analysis, the order in which variables are entered in a Cholesky decomposition is of importance. Given the temporal succession between variables, and the fact that we were interested in examining the etiology of change and continuity between T1 and T2, we entered T1 measures first in the bivariate Cholesky decomposition, followed by T2 measures.

## Supplementary Material

Supplement

## Figures and Tables

**Figure 7.**
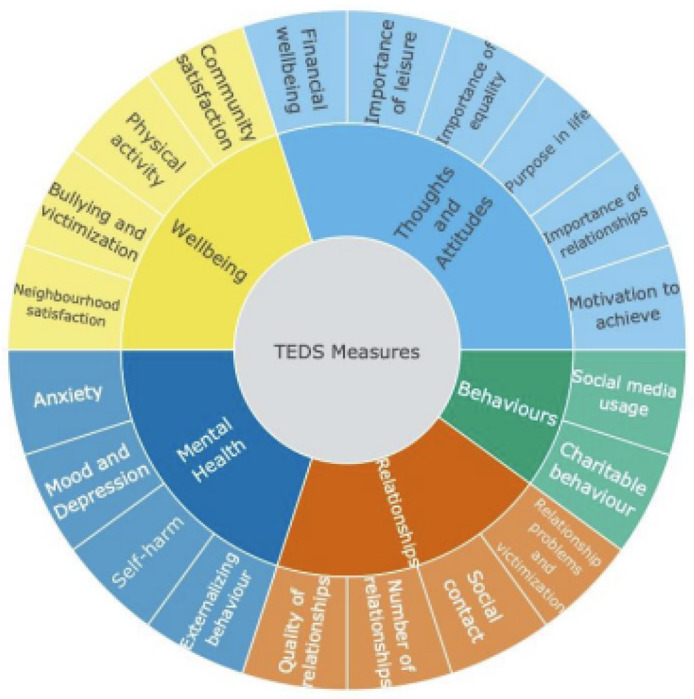
Summary of measures collected at T1 and T2

**Figure 8.**
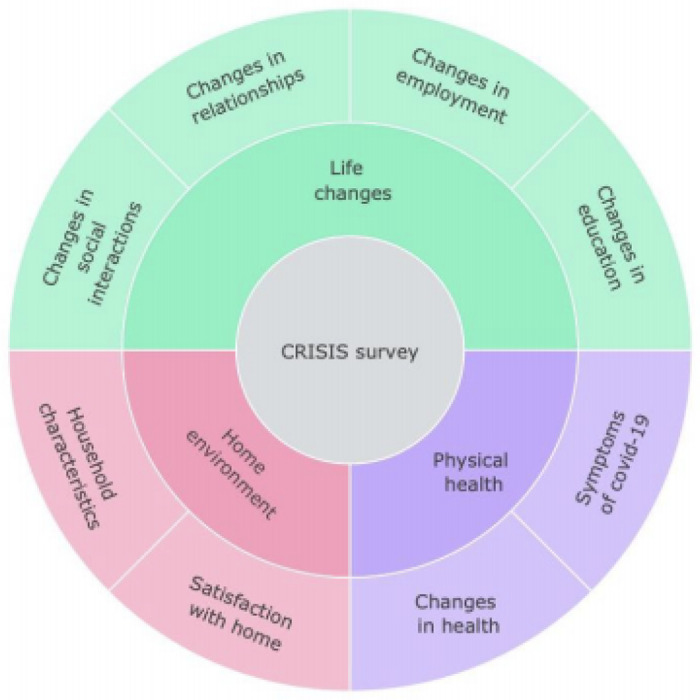
Summary of CRISIS survey collected at T1 and T2.

## References

[R1] Arnett J. J. (2014). Emerging Adulthood: The winding road from the late teens through the twenties. Second edition. Oxford University Press.

[R2] Boker S., Neale M., Maes H., Wilde M., Spiegel M., Brick T., … Fox J. (2011). OpenMx: an open source extended structural equation modeling framework. Psychometrika, 76, 306–317. 10.1007/s11336-010-9200-6 23258944 PMC3525063

[R3] Brooks S. K. (2020). The psychological impact of quarantine and how to reduce it: Rapid review of the evidence. The Lancet, 395, P912–920. 10.1016/S0140-6736(20)30460-8 PMC7158942 32112714

[R4] Cohen J. (1988). Statistical power analyses for the behavioral sciences. Second edition Hillsdale, NJ: Lawrence Erlbaum Associates.

[R5] Furr J. M., Comer J. S., Edmunds J. M., & Kendall P. C. (2010). Disasters and youth: A meta-analytic examination of posttraumatic stress. Journal of Consulting and Clinical Psychology, 78, 765–780. 10.1037/a0021482 21114340

[R6] Galea S., Merchant R. M., & Lurie N. (2020). The mental health consequences of COVID-19 and physical distancing: The need for prevention and early intervention. JAMA Internal Medicine. Published online 10 April 2020. doi:10.1001/jamainternmed.2020.1562 32275292

[R7] Harris J. R. (1998). The nurture assumption: Why children turn out the way they do. New York: The Free Press.

[R8] Knopik V. S., Neiderhiser J. M., DeFries J. C., & Plomin R. (2017). Behavioral genetics (7th ed.). New York: Worth.

[R9] McGue M., & Bouchard T. J. (1984). Adjustment of twin data for the effects of age and sex. Behavior Genetics, 14, 325–343. 10.1007/BF01080045 6542356

[R10] Merikangas K. & Stringaris A. (2020). The CoRonavIruS Health Impact Survey (CRISIS). Adult Self-Report Baseline Form Available from Published online 10 April 2020 argyris.stringaris@nih.gov.

[R11] Neale M. C., Boker S. M., Bergeman C. S., & Maes H. H. (2015). The utility of genetically informative data in the study of development In Methodological Issues in Aging Research. 10.4324/9781315820989-14

[R12] Plomin R. (2018/2019). Blueprint: How DNA Makes Us Who We Are. London: Allen Lane/Penguin.

[R13] Plomin R., & Daniels D. (1987). Why are children in the same family so different from each other? (With Open Peer Commentary and Response). Behavioral and Brain Sciences, 10, 1–60. doi: 10.1017/S0140525X00055941

[R14] Plomin R., DeFries J. C., Knopik V. S., & Neiderhiser J. M. (2016). Top 10 replicated findings from behavioral genetics. Perpectives on Psychological Science, 11, 3–23. doi: 10.1177/1745691615617439 PMC4739500 26817721

[R15] Polderman T. J. C. , (2015). Meta-analysis of the heritability of human traits based on fifty years of twin studies. Nature Genetics, 47, 702–709. 10.1038/ng.3285 25985137

[R16] Rijsdijk F. V, & Sham P. C. (2002). Analytic approaches to twin data using structural equation models. Briefings in Bioinformatics, 3(2), 119–133. 10.1093/bib/3.2.119 12139432

[R17] Rimfeld K., Malanchini M., Spargo T, Spickernell G., Selzam S., McMillan A., Dale P. S., Eley T. C., & Plomin R. (2019). Twins Early Development Study: A Genetically Sensitive Investigation into Behavioral and Cognitive Development from Infancy to Emerging Adulthood. Twin Research and Human Genetics, 22, 508–513. doi: 10.1017/thg.2019.56 31544730 PMC7056571

[R18] Smith C., Christoffersen K., & Davidson H. (2011). Lost in transition: The dark side of emerging adulthood. Oxford, UK: Oxford University Press.

